# Editorial: Advances in surgical approaches for the treatment of glioma

**DOI:** 10.3389/fonc.2023.1236341

**Published:** 2023-07-11

**Authors:** Hongmin Bai, Che Jiang

**Affiliations:** Department of Neurosurgery, General Hospital of Southern Theater Command, Guangzhou, Guangdong, China

**Keywords:** surgery, glioma, direct electrical stimulation (DES), IMRI, histological examination

Presently, maximal-safe resection is still the primary pursuit for surgically treating glioma. Accordingly, there are a variety of novel techniques being developed. Therefore, this Research Topic aimed to summarize advances in glioma surgery along with emerging auxiliary techniques.

On the whole, there are four aspects to consider: balancing between the extent of resection and preserving neurological function, shortening intraoperative examination time while improving the accuracy of detection, minimizing surgical incision while achieving total resection, and utilizing *in vivo* histological testing just before completion of resection while guaranteeing patient safety.

## 1 Clinical studies

Intraoperative direct electrical stimulation (DES) can directly identify neural networks crucial for brain function and remains the gold standard for eloquent cortex localization (1, 2). Although there remains some debate (3, 4), awake brain mapping plus DES is generally associated with more extensive resection, better overall survival, and fewer severe persistent neurological deficits compared with resection under general anesthesia (5–8). Wang et al. presented their experience in awake craniotomy with intraoperative DES mapping for gliomas invading eloquent areas. With a similar surgical strategy, Yao et al. reviewed their single-center experience of treating diffuse lower grade glioma (DLGG) in the central lobe (including precentral and postcentral gyri and paracentral lobule). Both studies reported favorable results. Moreover, Wang et al. established a high sensitivity of the Montreal Cognitive Assessment (MoCA), a relatively brief screening tool for assessment of this population’s cognitive impairment.

Compared with awake surgery, surgery under general anesthesia is less time-consuming, with lower intraoperative risk (9, 10). Cui et al. reported their experience of removing glioma under general anesthesia assisted by intraoperative multimodal techniques (combined use of neuronavigation, iMRI, with/without DES/neuromonitoring). The large sample size (nearly 500 patients) was the strength of this study. The researchers confirmed that the application of multimodal techniques improved the extent of resection (EOR) and rate of gross total resection (GTR) while associated with a lower incidence of permanent language deficits (PLD). They also found clinical factors for predicting language deficit. It is worth noting that the researchers achieved a higher rate of GTR (72.7%) than all previous studies of awake craniotomy, with only a slightly higher incidence of PLD (13.4%). The established predictive model may provide surgeons and patients a reference in choosing an appropriate surgical strategy (e.g., awake craniotomy with DES for patients with the highest risk of language deficit, while multimodal techniques under general anesthesia for those with moderate risk). However, the model should be validated with more studies.

Transcranial magnetic stimulation (TMS) can generate a magnetic field, inducing transient electric fields within the targeted brain cortex. High/low frequency stimulation excites/inhibits neuron activity (11). As another way to directly test the crucial neurocircuits of brain function besides DES, navigated TMS (nTMS) has a similar effect in predicting the location of eloquent areas (12–15). Li et al. verified the reliability of individual-target TMS in preoperative mapping. They showed that compared to intraoperative DES in awake surgery, the combination of nTMS can lead to an improvement in language performance as well as in brain-structure preservation. Precise localization by preoperative methods may help reduce the time required for awake surgery.

It has been reported that subtle neuropsychological disturbances are more frequent than traditional thoughts after glioma surgery (16–18). Therefore, some scholars even proposed performing awake surgery in all LGG patients regardless of tumor location and monitoring more subtle cognitive and behavioral functions intra-operatively (19). As studies showed mental disorders such as depression (20–22) and post-traumatic stress disorder (23, 24) were associated with shorter survival periods of LGG patients, the underlying neurocircuitry of emotion may also be considered.

Less traumatic procedures could reduce patients’ pain, improve cosmetic appearance, mitigate neurophysiological reflex response, and reduce the risk of infection (25, 26). Some previous studies have utilized an endoscope to resect deep-seated glioblastoma (27, 28). Sakata et al. further expanded its use in resecting superficial glioblastomas. In their six-case series, all achieved gross total/near total resection. The author emphasized the importance of prior endovascular tumor embolization. Huge intraoperative blood loss (>1000 ml) only occurred in one case, for whom the preceding embolization could not be performed due to the non-localization of a proper feeder artery.

## 2 Imaging studies

Jiang et al. reviewed four main intraoperative systems for delineating malignant tumors: intraoperative MRI (iMRI), fluorescence, Raman histology, and mass spectrometry. iMRI overcomes brain drift defects and has been shown to increase the complete resection rate (29, 30). Besides, functional MRI (fMRI), diffusion tensor imaging (DTI), and MR spectroscopy (MRS) can be applied with patients asleep for the whole surgical procedure, giving a panoramic view of the functional brain cortex, fiber tracts, and metabolic change levels. To solve the problem that the DTI reconstruction process relies on the experience of operators and is time-consuming, Yuan et al. developed an in-house software, “DiffusionGo.” It can reconstruct DTI automatically and quickly. The researchers demonstrated its efficacy in language function preservation for three patients who underwent surgical resection of gliomas. DiffusionGo is especially suitable for intraoperative use, and its adaptation to iMRI may be a future research direction. Although clinical implementation should be further established with a larger sample size, this software has presented promising value.

Yang et al. also mentioned in their review of surgical treatment options in gliomas, the augmented reality (AR) neuronavigation system, which integrates MR/CT images into the surgical field and presents three-dimensional virtual tissues to guide resection (31–33).

Cui et al. did bibliometric research on artificial intelligence developments in nervous system diseases. They found “glioma” to be the leading research hotspot, and “machine learning,” “brain metastasis,” and “gene mutations” were at the research frontier. This result indicated a promising prospect for radiomics biomarkers and multi-omics studies.

## 3 Histological studies

Hong et al. developed a new confocal laser endomicroscopy with a “Lissajous scanning pattern.” In *in vitro* and ex vivo experiments, they demonstrated its feasibility for indocyanine green (ICG) fluorescence-guided brain tumor diagnosis. Through direct tumor cell visualization, confocal laser endomicroscopy (CLE) can reveal tumor cell invasion in the adjacent normal brain and display the tumor-brain interface. Zhang et al. reviewed the latest research on Raman spectroscopy (RS) in glioma. RS is a label-free imaging method that uses intrinsic biochemical markers to identify tumors (34, 35) and has the advantages of being non-destructive, rapid and accurate. Presently, the above two techniques for histological tests are used for resected human tissue. Both methods can be equipped with miniaturized hand-held probes, so they can potentially test *in vivo* tumor tissue, offering guidance on the range of resection. However, their respective safety and accuracy should be further evaluated.

In summary, the 11 studies included in this Research Topic included advances in surgical approaches from various aspects, and future progress in this realm can be anticipated.

## Author contributions

HB and CJ wrote the manuscript. HB revised the manuscript. All authors contributed to the article and approved the submitted version.
